# Differential effects of peptidoglycan on colorectal tumors and intestinal tissue post-pelvic radiotherapy

**DOI:** 10.18632/oncotarget.12353

**Published:** 2016-09-30

**Authors:** Gen Li, Anqing Wu, Dandan Qi, Fengmei Cui, Yanan Zeng, Fang Xie, Hongya Wu, Yongping Gu, Qiu Chen, Xueguang Zhang

**Affiliations:** ^1^ School of Radiation Medicine and Protection, Soochow University, Suzhou 215123, P.R. China; ^2^ Stem Cell Research Laboratory of Jiangsu Province, Suzhou 215007, P.R. China; ^3^ Collaborative Innovation Center of Radiation Medicine of Jiangsu Higher Education Institutions, Soochow University, Suzhou 215123, P.R. China; ^4^ Department of Pathology, School of Biology & Basic Medical Science, Soochow University, Suzhou 215123, P.R. China; ^5^ Jiangsu Institute of Clinical Immunology, Suzhou 215007, P.R. China; ^6^ Experimental Centre of Medical College, Soochow University, Suzhou 215123, P.R. China

**Keywords:** peptidoglycan, colorectal tumor, intestine, pelvic radiotherapy, IL13-AKT3-mTOR pathway

## Abstract

Immediate medical intervention is required after pelvic tumor radiotherapy to protect the radiosensitive intestine and also to mitigate tumor growth. Toll-like receptors (TLRs) have been shown to promote tissue repair processes. Here, we analyzed the effect observed upon combining the TLR2 agonist, peptidoglycan (PGN), with radiation therapy on tumors as well as intestinal tissue, both in vitro and in vivo. In contrast to radiotherapy alone, PGN when combined with ionizing radiation (IR) elicited enhanced antitumor effects and also reduced the IR-induced intestinal damage. Mechanistic studies showed that PGN first induced an IL13 response in the irradiated intestine, but was decreased in tumor cell models screened by Th1/Th2 FlowCytomix assay and validated by the application of IL13 and anti-IL13 neutralizing antibodies. Next, PGN stimulated Akt3, but not Akt1/2, as was verified by AKT1/2/3 plasmid transfection assay and in AKT1/2/3 knockout mice in vivo. Akt3 expression was inhibited in 20 μg/mL PGN-treated tumor cells and in 1.5 mg/kg PGN-treated mouse tumor models. However, Akt3 was raised via IL13 in the irradiated intestine and human intestinal cell line after the same treatment. Finally, PGN activated mTOR via IL13/AKT3 in the intestine and restored intestinal structure and function. As an adjuvant to radiotherapy, PGN inhibited tumorigenesis by suppression of mTOR activity. To summarize, the IL13/AKT3/mTOR pathway was lessened in PGN-treated irradiated tumors but was raised in the normal intestine tissue. This distinct effect of PGN on normal and tumor tissues during pelvic radiotherapy suggests that PGN may be a promising adjuvant therapy to radiation.

## INTRODUCTION

About 300,000 patients with gynecologic, bladder, rectal, and prostate cancers undergo pelvic radiotherapy worldwide every year. However, nine out of ten patients develop a permanent change in their bowel habits following radiation [1] irrespective of the quantum of radiation dose [2]. Bowel frequency, loose or liquid stools, fecal incontinence, and the need for undergarment protection were significantly more frequent in radiotherapy patients [3]. The side effects of pelvic radiotherapy such as bleeding, fistula formation, bowel obstruction, and secondary malignancy are very grave [4]. There is a need to develop anti-tumor drugs having high efficacy, but minimal bowel toxicity.

Toll-like receptors (TLRs) are shown to promote tissue repair processes. Inhibitors of TLR3 [5], and agonists of other TLRs such as TLR4, 5, and 9 [6–9] have been shown to possess radio-protective effects.

The effect of TLRs on tumor cells is still unclear [10]. In some tumor types, TLRs promote tumor proliferation and survival, as seen with TLR9 agonists [11], Pam2 lipopeptides (TLR2/6 ligands) [12], and flagellin [13]. However, TLRs have also been shown to be directly involved in tumor apoptosis. For example, TLR5 activation by flagellin elicited an innate immune response, causing decreased tumorigenesis in breast cancer cells [14]. TLR2 inhibited the production of the inflammatory cytokine interleukin-18 (IL-18) and protected mice from diethylnitrosamine (DEN)-induced hepatocellular carcinoma [15]. Loxoribin, a TLR7 ligand, inhibited tumor growth in xenograft models of colon and lung cancers, and these anti-tumor effects were mediated by increased CD4^+^T cell proliferation and reversal of Treg-mediated immunosuppression via dendritic cells (DCs) [16]. TLR9 agonists, such as CpG oligodeoxynucleotides, are under clinical trials for the treatment of several hematopoietic and solid tumors [17]. In light of these dual functions, it is imperative to further explore and delineate the functional role of TLRs in tumorigenesis.

The possibility of combining radiation and immune-based therapies to achieve better microenvironmental protection and tumor immunogenicity has recently emerged [18]. A TLR7 agonist was shown to possess adjuvant activity when combined with local radiotherapy [19–21]. However, the authors did not describe the effect of the therapy on normal tissues. In contrast, a TLR9 agonist limited the efficacy of cancer radiotherapy [22, 23]. We have previously reported the protective effect of peptidoglycan (PGN) against toxicity induced by ionizing radiation (IR) [24]. However, the effect of PGN administration on the intestine and pelvic tumor during pelvic cancer radiotherapy has not been established.

## RESULTS

### Radiotherapy combined with PGN inhibited tumor growth more effectively

**Figure 1:**
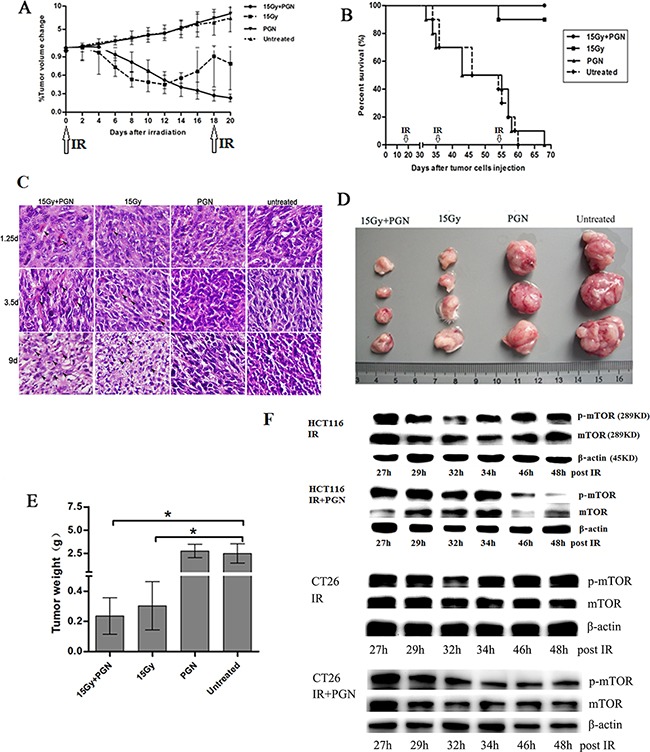
PGN synergizes with bowel irradiation to elicit enhanced antitumor responses in the CT26 model of colorectal cancer Mice received either PBS (n=19) or 1.5 mg/kg PGN (n=19) once every 19 days i.p., 15 Gy abdominal IR (n=20) given every 18 days or a combination of PGN and 15 Gy abdominal IR (n=20, PGN was given 24 h after IR). **A.** Tumor volumes were plotted from the first day of local radiotherapy (day 0). Growth curves are shown as a mean±standard deviation (n=6 per group, *p<0.05 relative to 15 Gy abdominal IR). **B.** Survival curves for above cohorts up to 70 days (n=10 per group, arrows indicate abdominal IR on days 0, 18, 36, and 54). **C.** H&E staining of tumors on 1.25, 3.5, and 9 days after IR. In these representative pictures, black arrows mark apoptotic cells. **D.** Tumor images at termination (15 Gy+PGN, n=4; 15 Gy, n=4; PGN, n=3; untreated, n=3). **E.** Tumor weights of above cohorts 9 days after the initial IR (*p<0.05 relative to untreated group). **F.** Western blots for mTOR and phospho-mTOR expression following 15 Gy IR or 15 Gy IR + 20 μg/ml PGN in HCT116 and CT26 cells, at indicated time points.

As shown in Figure 1A, tumor volumes increased steadily to approximately 5 times their initial size in both untreated and mice treated with PGN alone. As a monotherapy, PGN at a dose of 1.5 mg/kg per mouse had no effect on tumor burden or survival relative to the PBS control. However, 15 Gy local radiotherapy and 15 Gy + PGN treatment significantly reduced tumor size, suggesting that radiation has an inhibitory effect on tumor growth. The effects of 15 Gy radiation alone were transient, and tumor volumes began to increase 12 days after radiotherapy; however, the tumor volumes in the 15 Gy radiation + PGN-treated group continued to decrease, with significant differences between the radiation alone and radiation + PGN treatment groups at 16, 18, and 20 days after initial local irradiation (p<0.05, Figure 1A). An additional 15 Gy dose of radiation was administered on day 18, which also had an inhibitory effect on tumor growth. At day 70, all mice in the combination treatment group were alive, while 90% of mice treated with radiation alone were alive (Figure 1B). All untreated and mice treated with PGN alone died by day 70 (Figure 1B). Tumor sections at 1.25 and 3.5 days after IR showed morphological characteristics of apoptosis, specifically cell shrinkage and chromatin condensation (Figure 1C). Sections from mice that were irradiated showed more apoptotic and karyolitic cells than those that were not. On post-IR day 9, tumor weights from irradiated mice were less than 0.5 g, while tumors from mice that were not irradiated were more than 2.5 g (p<0.05, Figure 1D, 1E).

The downstream target of the phosphatidylinositol-3-kinase (PI3K) /AKT pathway is the mammalian target of rapamycin (mTOR). mTOR is a serine/threonine-specific protein kinase that boosts cell growth and proliferation. Irradiation of HCT116 cells transiently reduced expression of both mTOR and phosphorylated mTOR between 24 and 32 h; however, IR in conjunction with PGN treatment continuously decreased mTOR and phosphorylated mTOR expression from 24–48 h. Similar effects were observed in CT26 cells (Figure 1F).

### PGN can promote the recovery of intestinal structure and function after irradiation

**Figure 2:**
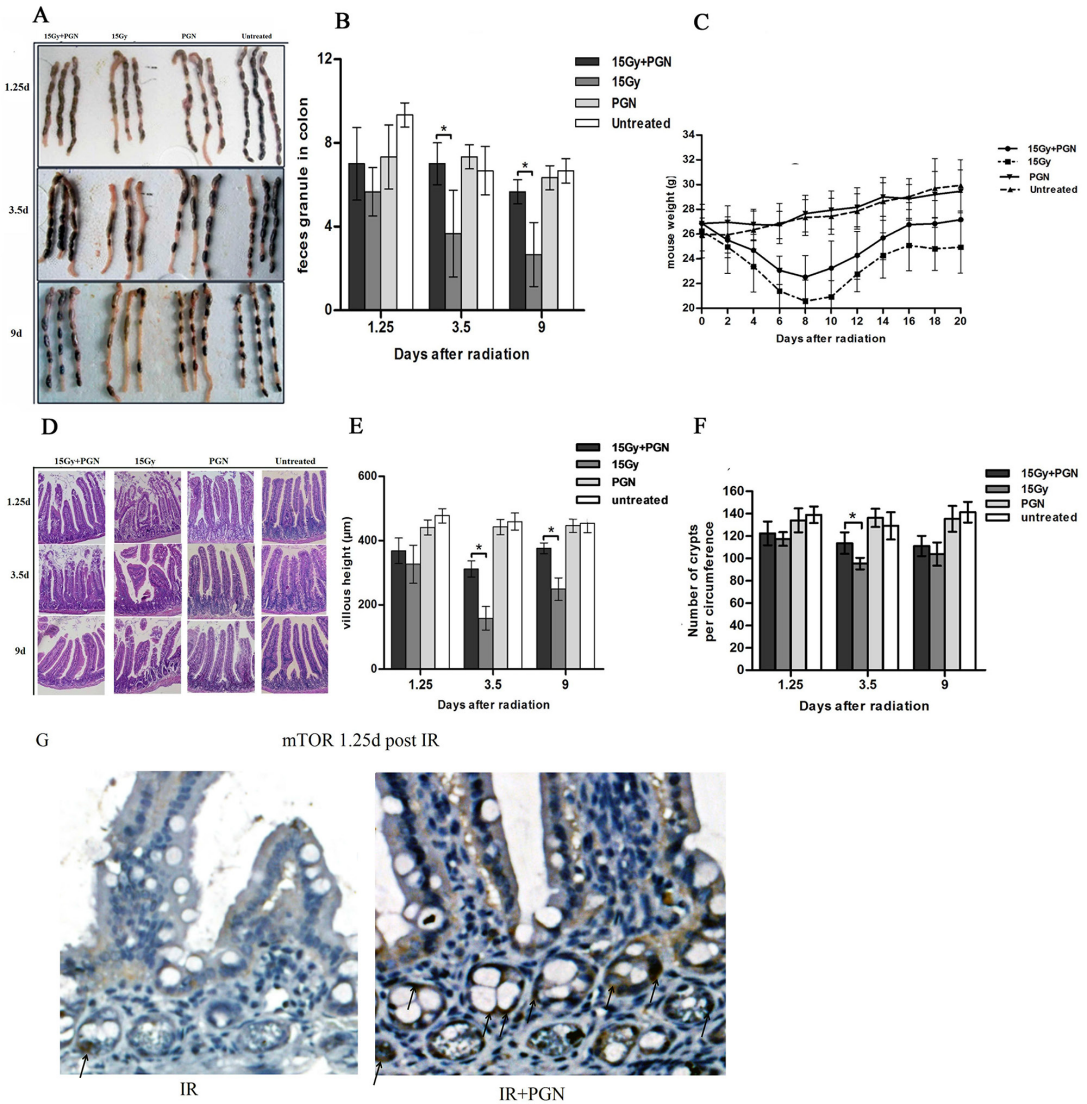
Peptidoglycan helped to sustain the structure and function of the irradiated intestine **A.** A representative image of stools in the colon. **B.** Feces discharged from the bowels were counted at 1.25, 3.5, and 9 days after IR (n=3 per group). **C.** Body weights after radiotherapy were recorded every other day in each group for a total of 20 days (n=6 per group). **D.** Representative images of the pathologic and morphologic changes of the small intestinal villi in each group at 1.25, 3.5, and 9 days after IR using H&E staining (200× magnification). **E.** Villi height of each group (n=3) were measured and compared at 1.25, 3.5, and 9 days after IR. **F.** Number of intestinal crypts along the jejunum circumference (10 circuits in total) in each group at 1.25, 3.5, and 9 days after IR was counted. Data are shown as the mean of 10 circuits±standard deviation. Asterisk indicates p<0.05. **G.** immunohistochemistry of mouse intestinal tissue 1.25 days after 15 Gy IR. Arrows indicate the mTOR positive crypts (400×).

Stool formation assessments were carried out in order to detect malabsorption and hypoperistalsis. The number of fecal particles in the colon did not significantly differ between the radiation-alone and radiation + PGN groups at 1.25 days after IR, but was significantly less at 3.5 and 9 days after IR in the radiation alone group (p<0.05). At 3.5 days after IR, the feces of the radiation-alone mice were soft, thick, and gray, but appeared hard and normal-colored in the radiation + PGN group (Figure 2A, 2B).

The body weight of mice in the PGN-alone and untreated groups did not change up to 20 days, but significantly decreased in the irradiated mice within 10 days after IR. In addition, mice exposed to radiation alone lost more weight in 20 days and recovered more slowly than irradiated mice who also received PGN (Figure 2C).

The untreated and PGN-alone treated mice had integrated villus epithelium and crypts. The intestinal epithelium of irradiated mice exhibited severe radiation damage 3.5 days after IR, as evidenced by eroded and truncated villi tips as well as significant necrosis of epithelial cells, vacuolization, and loose structure. Administration of PGN resulted in reduced intestinal damage at 3.5 days after IR, and the histology showed return to normalcy by day 9. The average lengths of villi in mice of the radiation-alone group were 158 μm and 250 μm, but were 312 μm and 376 μm at 3.5 and 9 days after IR, respectively, in the radiation + PGN group (p<0.05, Figure 2D, 2E).

H&E staining of the crypts of irradiated mice 3.5 days after IR showed sustained and significant crypt damage: elongated shape, deformation, and crypt numbers were reduced to approximately 96/circumference. Co-treatment with PGN resulted in intact crypts arranged in neat rows, with a significantly higher number of crypts/circumference (114, Figure 2F).

Immunochemistry analysis demonstrated that PGN induced mTOR expression after IR treatment (Figure 2G).

### PGN attenuated the effects of radiation on intestinal crypts

**Figure 3:**
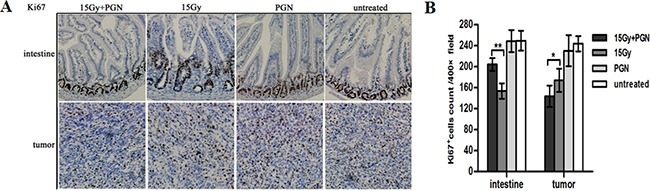
Contrasting effect of PGN treatment on the intestine and tumor following irradiation **A.** Representative images of Ki67 immunohistochemical staining of tissues of the jejunum and tumor at 3.5 days after local IR (200× magnification). **B.** Ki67-positive cells were counted in 10 unduplicated fields visualized at 400× magnification. Data are shown as the mean of 10 view fields±standard deviation (*, p<0.05; **, p<0.01).

The proliferative capacities of the intestines and tumors were measured by Ki67 staining at 3.5 days after irradiation. There were significantly more number of Ki67- positive cells in the intestines of the radiation + PGN group than in the radiation-alone group; however, fewer Ki67-positive cells were observed in the tumors of the radiation + PGN group than in the radiation alone group (p<0.05, Figure 3).

### PGN differentially regulated IL13 and TNF-α expression in the intestine and tumor 9 days after IR

**Figure 4:**
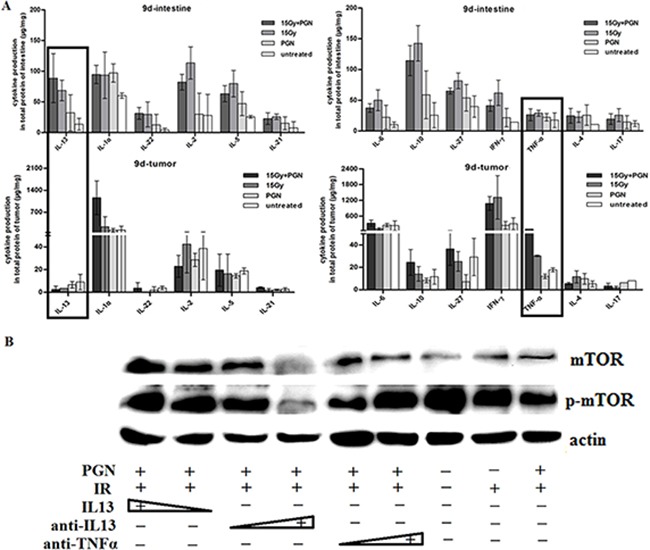
IL13 played a significant role in PGN's differential effect on irradiated intestine and tumor **A.** Flowcytomix assays using bead technology were utilized to detect cytokine production (μg cytokine/mg tissue weight) in intestinal and tumor tissues that were untreated, treated with PGN alone, radiation alone, or radiation + PGN at 9 days after IR. **B.** Western blot analysis of mTOR and phospho-mTOR expression in HCT116 cells following treatment with radiation alone or radiation + PGN in the presence or absence of IL13 (0.8 and 1.2 ng/mL), IL13 neutralizing antibody (0.12 and 0.2 μg/mL), or TNF-α neutralizing antibody (0.04 and 0.08 μg/mL).

The immune microenvironment influences the response to therapy. Intestinal epithelial cell (IEC) homeostasis and repair which is mediated through microbe-sensing, TLR-induced inflammatory pathways and inflammation-associated cancer development, is also influenced by inflammatory cytokines. As shown in Figure 4A, IL13, IL1α, IL22, IL2, IL5, IL21, IL6, IL10, IL27, IFNγ, TNFα, IL4, and IL17 cytokines were expressed in subsets of mice intestines and tumors 9 days after IR. Among these cytokines, TNF-α expression did not show any marked difference among intestines of mice of any of the treatment groups, but was increased in tumors of mice treated with radiation + PGN compared with untreated, PGN–alone, and radiation-alone treated mice. IL13 expression was significantly increased in irradiated small intestines; it was highest in the intestines treated with IR combined with PGN. However, IL13 tended to be lower in irradiated tumors as compared to untreated tissues; it was the lowest in tumors treated with IR and PGN.

Different concentrations of IL13, IL13 neutralizing antibody, and TNFα neutralizing antibody were applied to PGN + radiation-treated HCT116 cells. mTOR and phospho-mTOR expression increased with increasing concentrations of IL13. Increasing concentrations of IL13 neutralizing antibody decreased phospho-mTOR expression; however, addition of TNFα neutralizing antibody had the opposite effect. This suggested that PGN promoted the secretion of TNFα by tumors following irradiation and that TNFα plays a role in radiation-induced inhibition of tumor growth. TNFα expression did not change significantly in irradiated intestines, suggesting that PGN does not have a similar effect in the normal tissue (Figure 4B).

### Akt3 but not Akt1/2 plays an important role in PGN's differential effects on tumor and intestinal tissues after IR

**Figure 5:**
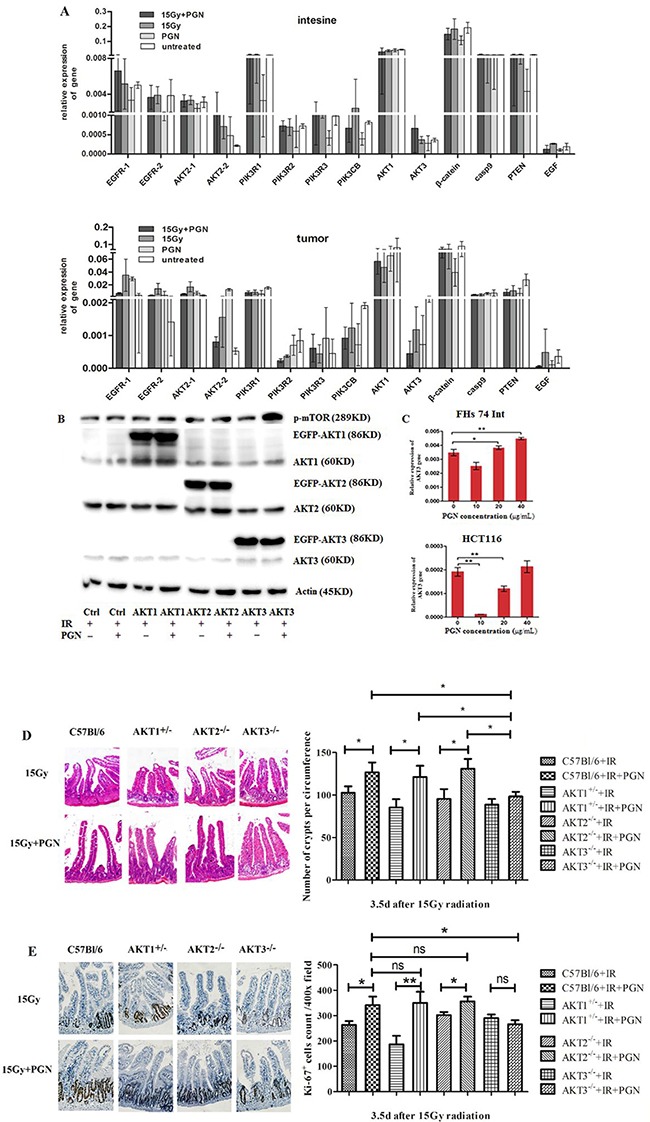
AKT3 was implicated in PGN's distinct effects on intestinal and tumor cell proliferation after IR **A.** EGFR (two transcripts), AKT2 (two transcripts), PIK3R1, PIK3R2, PIK3R3, β-catenin, AKT3, AKT1, Casp9, PTEN, PIK3CB, and EGF expression were detected by real-time PCR. Data are shown as the mean of 2^(Ct, actin-Ct, target)^±standard deviation. **B.** Western blots for AKT1/2/3 and p-mTOR in AKT1/2/3 overexpressing HCT116 cells. ‘EGFP-AKT' represents exogenously expressed AKT whereas ‘AKT’ represents endogenously expressed protein. β-actin was used as loading control. **C.** Real-time PCR analysis of AKT3 expression in FHS 74 Int and HCT116 cells following treatment with 0, 10, 20, and 40 μg/mL PGN (*, p<0.05; **, p<0.01). **D.** H&E staining of the cross section intestines of AKT1^+/−^, AKT2^−/−^, AKT3^−/−^, and control C57Bl/6 mice, irradiated or treated with PGN after IR. The crypts per circumference were counted. PGN had no effect on the number of crypts in irradiated AKT3^−/−^ mice. **E.** Ki67 immunohistochemical staining showed that the number of Ki67^+^ crypt did not increase when irradiated AKT3^−/−^ mice were treated with PGN. n=6 in each group; magnification: 400×. *, p <0.05; **, p <0.01.

Expression of a series of proliferation-related genes was investigated in tumor and intestinal tissues by real-time PCR. These included EGFR (two transcripts: EGFR-1,2), AKT2 (two transcripts: AKT2-1,2), PIK3R1, PIK3R2, PIK3R3, β-catenin, AKT3, AKT1, Casp9, PTEN, PIK3CB, and EGF. Within the AKT family, the expression of AKT1 was highest and AKT3 was lowest in both the intestines and the tumors. AKT1 and AKT2 transcript variant 1 (AKT2-1) expressions did not change markedly, but AKT2 transcript variant 2 (AKT2-2) and AKT3 levels were significantly increased in the intestines but were decreased in the tumors of mice treated with PGN + radiation compared to radiation alone (Figure 5A). However, AKT2-2 expression was lowest in the untreated tumors among all four groups. These results suggest that AKT3 may be a key player in PGN's differential biological effects on intestinal and tumor tissues following IR.

Western blot analyses further confirmed these results, with exogenous overexpression of AKT3 (EGFP-AKT3), but not AKT1/2 (EGFP-AKT1/2), increasing phospho-mTOR expression in PGN + radiation-treated HCT116 cells (Figure 5B).

FHs 74 Int small intestine epithelial cells and HCT116 colorectal carcinoma cells were used to represent intestinal and tumor tissues, respectively. Akt3 expression was detected in irradiated cells treated with 10, 20, or 40 μg/mL PGN. As shown in Figure 5C, 10 μg/mL PGN significantly decreased Akt3 expression in irradiated FHs 74 Int and irradiated HCT116 cells. At increasing PGN concentrations, expression of Akt3 increased in irradiated FHs 74 Int and irradiated HCT116 cells. Akt3 expression in irradiated FHs 74 Int treated with 20 μg/mL PGN was higher than in cells treated with radiation alone; however, this effect was not observed in irradiated HCT116 cells. Irradiated HCT116 cells treated with 40 μg/mL PGN closely resembled irradiated FHs 74 Int cells (Figure 5C). PGN at 20 μg/mL was selected for the treatment of cells.

AKT1^−/−^ mice die shortly after birth. Therefore, we utilized AKT1^+/−^ mice in these studies. The intestinal crypts of irradiated AKT1^+/−^ mice and AKT2^−/−^ mice treated with PGN had features consistent with those of irradiated C57Bl/6 mice, but the crypts of irradiated AKT3^−/−^ mice did not proliferate significantly following PGN treatment, as shown by Ki67 staining (Figure 5D, 5E).

### IL13 stimulates AKT3 expression

**Figure 6:**
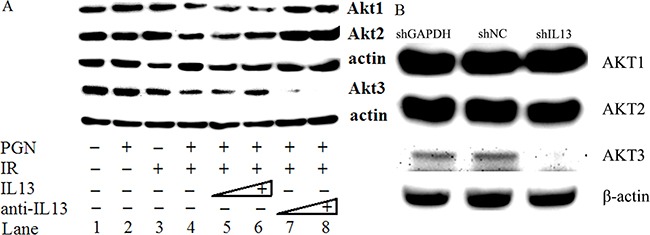
IL13 preferentially stimulated AKT3 **A.** Western blot analysis of AKT1/2/3 expression in untreated, PGN alone-treated, radiation alone-treated, or radiation + PGN treated HCT116 cells. Lanes 5 and 6 reflect addition of 0.8 and 1.2 ng/mL IL13, respectively. The concentrations of IL13 neutralizing antibody in lanes 7 and 8 were 0.12 and 0.2 μg/mL, respectively. β-actin was used as a loading control. **B.** shIL13/shNC/shGAPDH plasmids were transfected into HCT116 cells, as indicated. AKT1/2/3 was detected by western blot after 48 h. β-actin was used as a loading control.

As shown in Figure 6, irradiated HCT116 cells were treated with PGN and increasing concentrations of IL13. Akt2 and Akt3 expression increased at the higher IL13 concentrations. Although the addition of IL13 neutralizing antibody increased Akt2 expression even further, Akt3 expression was reduced (Figure 6A), suggesting that IL13 activated Akt3 but not Akt1 or Akt2 expression. Akt3 expression was abrogated when IL13 was knockdown by shIL13 (Figure 6B).

## DISCUSSION

After IR treatment with PGN, a TLR2 agonist, resulted in an enhanced antitumorigenic effect as compared to radiotherapy alone. Moreover, PGN mitigated the intestinal toxicity induced by IR. This effect was through differential production of IL13 and amplified via Akt3 and mTOR.

The dose of PGN was a key determinant of this effect. Akt3 was lowered in HCT116 cells but was markedly increased in FHs 74 Int cells upon treatment with 20 μg/ml PGN. A dose of 1.5 mg/kg of PGN in vivo induced intestinal proliferation and colorectal tumor suppression. According Fichera and Giese's [25], about 200 μg of PGN was required to saturate the binding sites in 1 μg of membranes. PGN may bind to the membrane receptor sites of the susceptible cells. Thereafter, the large amounts of PGN captured by the membranes may no longer be available for binding and inhibition of other cells, leading to their survival and proliferation.

Schaub et al. [26] have previously reported that pulmonary administration of PGN resulted in IL13 secretion *in vivo*. Ruíz-González et al. [27] also showed that keratinocytes treated with PGN increased IL13 production. Stimulation of IL13, a Th2 immune cytokine, led to an increase in crypt cell proliferation and goblet cell size and number via activation of PI3-kinase/AKT [28]. Additionally, Farmer et al. [29] reported that IL13 could protect mouse intestine from ischemia and reperfusion injury, and IL13 has been shown to promote colon carcinoma cell survival in a PI3-kinase dependent manner [30]. In this study, both PGN and IR treatments stimulated intestinal IL13, with combined PGN and IR treatment causing the highest IL13 production. Both PGN and IR decreased IL13 production in the tumor, with the lowest level detected in the PGN + IR-treated tumor.

Apart from the differences in the expression of IL13 in the small intestine and tumor, IL13 also has two kinds of receptor [31]. IL4Rα and IL13Rα1 dimerize to form the receptor, which binds IL13 with high affinity [32] and the complex mediates signal transduction through the JAK/STAT6 pathway [33]. Apart from IL13Rα1, IL13 has another cognate receptor, IL13Rα2, which binds IL13 with a markedly high affinity, although it lacks any significant cytoplasmic domain and, therefore, does not mediate signal transduction [34, 35]. It was reported that IL13Rα2 may suppress IL13 signal transduction through internalization of IL13 [36] and that the extracellular domain of IL13Rα2 may serve as a decoy receptor for IL13 [37, 38]. IL13Rα2 is highly expressed in several human tumors such as colorectal cancer [39] but is absent in normal tissues such as intestinal epithelium. IL13-mediated epithelial architectural and functional effects were dependent on the IL4R/IL13Rα1 signaling pathway but were IL13Rα2-independent 40]. Both IL13 dose and receptors demonstrated that IL13 signal transduction was higher in the intestine but lower in colorectal cancer.

The AKT family comprises three highly homologous kinases that suppress apoptosis and stimulate proliferation. However, the individual Akt isoforms have distinct downstream signaling functions in both normal tissues [41–47] and tumors depending on the cell type [48–53]. In our study, effects observed upon treatment of HCT116 cells with IL13 and IL13 neutralizing antibody suggested that Akt3 was the only family member targeted by IL13. Akt3 significantly activated the downstream mTOR kinase signaling. mTOR is known to stimulate cell growth and proliferation through regulation of ribosomal biogenesis and mRNA translation [54].

In conclusion, PGN has distinct biological effects on irradiated intestinal tissues and colorectal tumors via the IL13-Akt3-mTOR pathway. This pathway was inhibited in irradiated tumors but activated in irradiated intestines.

The role PGN in enhancing the radiation-induced antitumorigenic effect and in reducing the side effects of radiotherapy to the intestine is clinically relevant, and could potentially alter the current standard-of-care of colorectal cancer patients to include TLR2 agonists. With decreased radiotoxicity and increased antitumor activity, a larger therapeutic window could be established, which may result in better patient outcomes.

## MATERIALS AND METHODS

### Cell culture and treatment

CT26.WT is a BALB/c colon carcinoma cell line that was maintained in RPMI1640 medium supplemented with 10% fetal bovine serum (FBS). FHs 74. Int, a normal human intestinal cell line, was purchased from ATCC and was cultured in Hybri-Care Medium ATCC 46-X supplemented with 30 ng/ml epidermal growth factor (EGF, Peprotech, NJ, USA) and 10% FBS. HCT116 is a human colorectal carcinoma cell and was cultured in DMEM medium supplemented with 10% FBS. All cells were incubated at 37°C and 5% CO_2_. Both FHs 74. Int and HCT116 cells were treated with 10, 20, and 40 μg/mL PGN (from *Staphylococcus aureus*, Sigma, St. Louis, MO, USA). PGN was used at 20 μg/mL in all other *in vitro* experiments. HCT116 cells were treated with 20 μg/mL of PGN alone, 15 Gy irradiation alone, 15 Gy irradiation followed by 20 μg/mL PGN at 24 h, 15 Gy irradiation followed by 0.8 or 1.2 ng/mL IL13 (Peprotech) 2 h prior to 20 μg/mL of PGN at 24 h, 15 Gy irradiation followed by 0.12 or 0.2 μg/mL anti-IL13 (Peprotech) 2 h prior to 20 μg/mL of PGN at 24 h, or 15 Gy irradiation followed by 0.04 or 0.08 μg/mL anti-TNFα (Peprotech) 2 h prior to 20 μg/mL of PGN at 24 h.

### Established *in vivo* Matrigel-tumor growth assays and treatment

All animal studies were performed in accordance with the Animal Care Guidelines of Soochow University. Five- to seven-week-old male BABL/c mice (SLACCAS, Shanghai, China) were kept in animal maintenance facilities under conditions of controlled illumination (12:12 h light/dark cycle), humidity (30–50%), and temperature (18–22°C) and were fed a normal rodent laboratory diet and water. Mice (112 total) bearing BABL/c colon carcinoma at left abdominal derived from Matrigel (Becton Dickinson, San Jose, CA) suspensions 10^6^ CT26.WT cells (ATCC, Manassas, VA) were used. Mouse weights and tumor volume were determined using caliper measurements and the formula volume (mm^3^) = (length*width^2^)/2.

In the untreated group, 100 μl PBS was administered. In the pharmacotherapy group, an injection of 1.5 mg/kg PGN (1.5 mg/kg) was administered intraperitoneally (i.p). High-dose hypofractionated radiotherapy was adopted so as to reduce the frequency of animals were anesthetized and favor to observe intestinal damage. Irradiation (15 Gy) of the abdomen was performed every 18 days on anesthetized mice (i.p. administration of 0.36% chloral hydrate at 0.8 mL/100 g body weight) using a Philips SL18 X-ray system (9 MeV electron beam irradiation, Redhill, UK) at a dose rate of 200 cGy/min following the biosafety guidelines observed in China. For combination treatments, 15 Gy irradiation of the abdomen was followed by i.p. administration of 1.5 mg/kg PGN at 24 h. Following irradiation, mice were returned to cages (4 mice/cage) and were given free access to food and water. Ten mice per group were used for recording body weight, tumor size and survival studies every two days.

Anesthetized C57Bl/6, AKT1^+/−^, AKT2^−/−^, and AKT3^−/−^ mice (6–8 weeks, male, n=12 each, Model Animal Research Center of Nanjing University, Nanjing, China) underwent 15 Gy irradiation of the abdomen. Half of these mice were also treated with 1.5 mg/kg PGN 24 h after irradiation. Intestines were harvested and analyzed at 3.5 days after irradiation.

### Vector construction and transfection

Full length coding sequences of Akt1, 2, 3 genes were cloned and inserted into the pEGFP-C3 vector (Clontech, Mountain View, CA, USA) and transfected into HCT116 cells via DNA Transfection Reagent (Biotool, Houston, TX, USA) per the manufacturer's instructions. Cells were exposed to 15 Gy irradiation 24 h after transfection and half of these cells were treated with 20 μg/mL PGN 48 h after transfection. All cells were collected 3, 5, 8, 10, 22, and 24 hours after PGN treatment.

IL13 RNAi sequence (5'-AATGGCAGCATGGT ATGGAG-3') was inserted into pGPU6/GFP/Neo vector (GenePharma, Shanghai, China) and transfected into HCT116 cells in parallel with shNC (negative control) and shGAPDH plasmids. Forty-eight hours after transfection, proteins were extracted.

### Assays for stool formation

BALB/c mice were sacrificed 1.25, 3.5, and 9 days after IR and the entire colon starting from the anus was harvested. Loose, yellow content in the lumen was defined as poor stool formation or diarrhea, while solid, dark, granulated content was defined as formed stool.

### Determination of mRNA expression by real-time polymerase chain reaction (qPCR)

At select time points following treatment, RNA was isolated using Trizol (Invitrogen, Grand Island, NY, USA). Reverse transcription (RT) was performed according to the manufacturer's instructions (Moloney Murine Leukemia Virus Reverse Transcriptase cDNA Synthesis Kit, Takara, Otsu, Shiga, Japan) using 1 μg of RNA in a 20 μL reaction volume. The cDNA amplification and analysis was performed using real-time PCR (ABI Prism 7500, Applied Biosystems, GmbH, Darmstadt, Germany). TaqMan primers and probes for EGFR (epidermal growth factor receptor, two transcripts), AKT2 (thymoma viral proto-oncogene 2, two transcripts), PIK3R1 (phosphoinositide-3-kinase, regulatory subunit 1 (alpha)), PIK3R2 (phosphoinositide-3-kinase, regulatory subunit 2 (beta)), PIK3R3 (phosphoinositide-3-kinase, regulatory subunit 3 (gamma)), β-catenin ((cadherin associated protein), beta 1), AKT3 (thymoma viral proto-oncogene 3), AKT1 (thymoma viral proto-oncogene 1), Casp9 (caspase 9), PTEN (phosphatase and tensin homolog), PIK3CB (phosphatidylinositol-4,5-bisphosphate 3-kinase, catalytic subunit beta), EGF (epidermal growth factor) were purchased from Roche Diagnostics Corporation (Indianapolis, IN, USA). The cDNA was denatured at 95°C for 30 s followed by 40 cycles of 95°C for 5 s and 55°C for 20 s. The comparative *C*t (threshold cycles, Δ*C*t) quantification method was used to quantify the different target genes after normalizing with β-actin.

### Western blot analysis

Total cell lysates (20 μg) were separated using sodium dodecyl sulfate (SDS)-10% polyacrylamide gel electrophoresis (PAGE) and were transferred to Immobilon-P membranes (Millipore, Bedford, MA, USA). Membranes were blocked in 5% non-fat milk and incubated with primary antibodies overnight at 4°C. Membranes were then washed and incubated with horseradish peroxidase-conjugated secondary antibodies (anti-mouse or anti-rabbit) at room temperature for 2 h. Proteins were visualized using SuperSignal West Pico Luminol/Enhancer solution (Thermo Fisher Scientific Inc., Rockford, IL, USA). Akt1,2,3 primary antibody (Cell Signaling, Danvers, MA, USA) was diluted 1:200 in 0.1% milk/Tris-buffered saline and Tween-20 (TBS-T). β-actin (Bioworld Technology Inc., St. Louis Park, MN, USA), mTOR, and phospho-mTOR (Abcam, Cambridge, MA, USA) primary antibodies were all diluted 1:500 in TBS-T. Anti-mouse or anti-rabbit secondary antibodies (1 mg/mL, Dako North America Inc., Carpinteria, CA, USA) were diluted 1:1,000 in 5% milk/TBS-T.

### Haematoxylin and eosin (H&E) staining and immunohistochemistry

Tissues (tumor and normal intestine) were collected from mice and fixed in formalin solution. Tissue alterations were evaluated by histological analysis after H&E staining. For intestines, at least 20 villi were measured by length in each mouse. Complete crypts were also counted.

For immunohistochemical staining, paraffin blocks were cut into 4-μm sections that were mounted, deparaffinized in xylene, and rehydrated in decreasing concentrations of ethanol. Antigen retrieval was performed using citrate buffer, heating sections in a pressure cooker for 5 min and subsequently cooling to room temperature. Blocking of endogenous peroxidases was accomplished by incubating sections in 3% hydrogen peroxide for 5 min. Ki67 (BD Pharmingen, San Diego, CA, USA, 1:200)/mTOR (Cell Signaling, 1:100) antibody was incubated with sections overnight at 4°C. Immunostaining was performed using an Envision System and diaminobenzidine visualization (Dako) according to the manufacturer's instructions. Sections were counterstained with haematoxylin for 1 min, rinsed in water, dehydrated in increasing concentrations of ethanol followed by clearance with xylene, and cover-slipped permanently for light microscopy.

### Measurement of pro-inflammatory molecules

Tumors and intestines from three mice per group were used in this experiment. After tissues were grinded and lysed (lysis buffer from Cell Signaling, proteinase inhibitor from Roche), proteins were quantified using Bicinchoninic Acid Protein Assay Reagent (Thermo Fisher). Flowcytomix assays using bead technology (0.025–0.05 mL per assay for several cytokines) were adopted to determine the levels of multiple cytokines 9 days after IR. Mouse Th1/Th2 10plex FlowCytomix for granulocyte-macrophage colony-stimulating factor (GM-CSF); interferon (IFN)-γ; interleukin (IL)1, IL2, IL4, IL5, IL6, IL10, and IL17; tumor necrosis factor (TNF)α; and mouse IL21, IL27, IL22, and IL13 FlowCytomix Simplex were purchased from Bender Medsystems (eBioscience, San Diego, CA, USA) and assessed using a flow cytometer (BD FACSCalibur, BD Biosciences, Franklin Lakes, NJ).

### Statistical analyses

The data were analyzed using Windows SPSS version 10.0 (SPSS Inc., Chicago, IL, USA). Statistical analyses were performed using a Student's t-test. The data are presented as mean ± standard deviation. Survival data were assessed using Kaplan–Meier analysis. A p-value <0.05 was considered statistically significant.

## References

[1] Andreyev HJN (2007). Gastrointestinal Problems after Pelvic Radiotherapy: the Past, the Present and the Future. Clinical Oncology.

[2] Sermeus A, Leonard W, Engels B, De Ridder M (2014). Advances in radiotherapy and targeted therapies for rectal cancer. World J Gastroenterol.

[3] Andreyev HJN (2005). The gastrointestinal complications of pelvic radiotherapy: are they of any importance?. Gut.

[4] Andreyev J (2007). Gastrointestinal symptoms after pelvic radiotherapy: a new understanding to improve management of symptomatic patients. Lancet Oncol.

[5] Takemura N, Kawasaki T, Kunisawa J, Sato S, Lamichhane A, Kobiyama K, Aoshi T, Ito J, Mizuguchi K, Karuppuchamy T, Matsunaga K, Miyatake S, Mori N (2014). Blockade of TLR3 protects mice from lethal radiation-induced gastrointestinal syndrome. Nat Commun.

[6] Burdelya LG, Krivokrysenko VI, Tallant TC, Strom E, Gleiberman AS, Gupta D, Kurnasov OV, Fort FL, Osterman AL, Didonato JA, Feinstein E, Gudkov AV (2008). An agonist of toll-like receptor 5 has radioprotective activity in mouse and primate models. Science.

[7] Burdelya LG, Brackett CM, Kojouharov B, Gitlin II, Leonova KI, Gleiberman AS, Aygun-Sunar S, Veith J, Johnson C, Haderski GJ, Stanhope-Baker P, Allamaneni S, Skitzki J (2013). Central role of liver in anticancer and radioprotective activities of Toll-like receptor 5 agonist. Proc Natl Acad Sci U S A.

[8] Lacave-Lapalun JV, Benderitter M, Linard C (2014). Flagellin and LPS each restores rat lymphocyte populations after colorectal irradiation. J Leukoc Biol.

[9] Saha S, Bhanja P, Liu L, Alfieri AA, Yu D, Kandimalla ER, Agrawal S, Guha C (2012). TLR9 agonist protects mice from radiation-induced gastrointestinal syndrome. PLoS One.

[10] Matijevic T, Pavelic J (2010). Toll-like receptors: cost or benefit for cancer?. Curr Pharm Des.

[11] Herrmann A, Cherryholmes G, Schroeder A, Phallen J, Alizadeh D, Xin H, Wang T, Lee H, Lahtz C, Swiderski P, Armstrong B, Kowolik C, Gallia GL (2014). TLR9 is critical for glioma stem cell maintenance and targeting. Cancer Res.

[12] Yamazaki S, Okada K, Maruyama A, Matsumoto M, Yagita H, Seya T (2011). TLR2-dependent induction of IL-10 and Foxp3+ CD25+ CD4+ regulatory T cells prevents effective anti-tumor immunity induced by Pam2 lipopeptides in vivo. PLoS One.

[13] Song EJ, Kang MJ, Kim YS, Kim SM, Lee SE, Kim CH, Kim DJ, Park JH (2011). Flagellin promotes the proliferation of gastric cancer cells via the Toll-like receptor 5. Int J Mol Med.

[14] Cai Z, Sanchez A, Shi Z, Zhang T, Liu M, Zhang D (2011). Activation of Toll-like receptor 5 on breast cancer cells by flagellin suppresses cell proliferation and tumor growth. Cancer Res.

[15] Li S, Sun R, Chen Y, Wei H, Tian Z (2015). TLR2 Limits Development of Hepatocellular Carcinoma by Reducing IL18-Mediated Immunosuppression. Cancer Res.

[16] Wang C, Zhou Q, Wang X, Wu X, Chen X, Li J, Zhu Z, Liu B, Su L (2015). The TLR7 agonist induces tumor regression both by promoting CD4^+^T cells proliferation and by reversing T regulatory cell-mediated suppression via dendritic cells. Oncotarget.

[17] Murad YM, Clay TM (2009). CpG oligodeoxynucleotides as TLR9 agonists: therapeutic applications in cancer. BioDrugs.

[18] Roses RE, Xu M, Koski GK, Czerniecki BJ (2008). Radiation therapy and Toll-like receptor signaling: implications for the treatment of cancer. Oncogene.

[19] Demaria S, Vanpouille-Box C, Formenti SC, Adams S (2013). The TLR7 agonist imiquimod as an adjuvant for radiotherapy-elicited in situ vaccination against breast cancer. Oncoimmunology.

[20] Adlard AL, Dovedi SJ, Telfer BA, Koga-Yamakawa E, Pollard C, Honeychurch J, Illidge TM, Murata M, Robinson DT, Jewsbury PJ, Wilkinson RW, Stratford IJ (2014). A novel systemically administered Toll-like receptor 7 agonist potentiates the effect of ionizing radiation in murine solid tumor models. Int J Cancer.

[21] Dovedi SJ, Melis MH, Wilkinson RW, Adlard AL, Stratford IJ, Honeychurch J, Illidge TM (2013). Systemic delivery of a TLR7 agonist in combination with radiation primes durable antitumor immune responses in mouse models of lymphoma. Blood.

[22] Kortylewski M, Pal SK (2014). The dark side of Toll-like receptor signaling: TLR9 activation limits the efficacy cancer radiotherapy. Oncoimmunology.

[23] Gao C, Kozlowska A, Nechaev S, Li H, Zhang Q, Hossain DM, Kowolik CM, Chu P, Swiderski P, Diamond DJ, Pal SK, Raubitschek A, Kortylewski M (2013). TLR9 signaling in the tumor microenvironment initiates cancer recurrence after radiotherapy. Cancer Res.

[24] Liu W, Chen Q, Wu S, Xia X, Wu A, Cui F, Gu YP, Zhang X, Cao J (2015). Radioprotector WR-2721 and mitigating peptidoglycan synergistically promote mouse survival through the amelioration of intestinal and bone marrow damage. J Radiat Res.

[25] Fichera GA, Giese G (1994). Non-immunologically-mediated cytotoxicity of Lactobacillus casei and its derivative peptidoglycan against tumor cell lines. Cancer Lett.

[26] Schaub B, Bellou A, Gibbons FK, Velasco G, Campo M, He H, Liang Y, Gillman MW, Gold D, Weiss ST, Perkins DL, Finn PW (2004). TLR2 and TLR4 stimulation differentially induce cytokine secretion in human neonatal, adult, and murine mononuclear cells. J Interferon Cytokine Res.

[27] Ruíz-González V, Cancino-Diaz JC, Rodríguez-Martínez S, Cancino-Diaz ME (2009). Keratinocytes treated with peptidoglycan from Staphylococcus aureus produce vascular endothelial growth factor and its expression is amplified by the subsequent production of interleukin-13. Int J Dermatol.

[28] Wang ML, Keilbaugh SA, Cash-Mason T, He XC, Li L, Wu GD (2008). Immune-mediated signaling in intestinal goblet cells via PI3-kinaseand AKT-dependent pathways. Am J Physiol Gastrointest Liver Physiol.

[29] Farmer DG, Ke B, Shen XD, Kaldas FM, Gao F, Watson MJ, Busuttil RW, Kupiec-Weglinski JW (2011). Interleukin-13 protects mouse intestine from ischemia and reperfusion injury through regulation of innate and adaptive immunity. Transplantation.

[30] Wright K, Kolios G, Westwick J, Ward SG (1999). Cytokine-induced Apoptosis in Epithelial HT-29 Cells Is Independent of Nitric Oxide Formation. J Biol Chem.

[31] Zurawski SM, Vega F, Huyghe B, Zurawski G (1993). Receptors for interleukin-13 and interleukin-4 are complex and share a novel component that functions in signal transduction. EMBO J.

[32] Hilton DJ, Zhang JG, Metcalf D, Alexander WS, Nicola NA, Willson TA (1996). Cloning and characterization of a binding subunit of the interleukin 13 receptor that is also a component of the interleukin 4 receptor. Proc Natl Acad Sci USA.

[33] O'shea JJ, Gadina M, Schreiber RD (2002). Cytokine signaling in 2002: new surprises in the Jak/Stat pathway. Cell.

[34] Kawakami K, Taguchi J, Murata T, Puri RK (2001). The interleukin-13 receptor alpha2 chain: an essential component for binding and internalization but not for interleukin-13-induced signal transduction through the STAT6 pathway. Blood.

[35] Zhang JG, Hilton DJ, Willson TA, McFarlane C, Roberts BA, Moritz RL, Simpson RJ, Alexander WS, Metcalf D, Nicola NA (1997). Identification, purification, and characterization of a soluble interleukin (IL)-13-binding protein. Evidence that it is distinct from the cloned Il-13 receptor and Il-4 receptor alpha-chains. J Biol Chem.

[36] Andrews AL, Nasir T, Bucchieri F, Holloway JW, Holgate ST, Davies DE (2006). IL-13 receptor alpha 2: a regulator of IL-13 and IL-4 signal transduction in primary human fibroblasts. J Allergy Clin Immunol.

[37] Chiaramonte MG, Mentink-Kane M, Jacobson BA, Cheever AW, Whitters MJ, Goad ME, Wong A, Collins M, Donaldson DD, Grusby MJ, Wynn TA (2003). Regulation and function of the interleukin 13 receptor alpha 2 during a T helper cell type 2-dominant immune response. J Exp Med.

[38] Rahaman SO, Sharma P, Harbor PC, Aman MJ, Vogelbaum MA, Haque SJ (2002). IL-13R (alpha) 2, a decoy receptor for IL-13 acts as an inhibitor of IL-4-dependent signal transduction in glioblastoma cells. Cancer Res.

[39] Zhou R, Qian S, Gu X, Chen Z, Xiang J (2013). Interleukin-13 and its receptors in colorectal cancer (Review). Biomed Rep.

[40] Wu D, Ahrens R, Osterfeld H, Noah TK, Groschwitz K, Foster PS, Steinbrecher KA, Rothenberg ME, Shroyer NF, Matthaei KI, Finkelman FD, Hogan SP (2011). Interleukin-13 (IL-13)/IL-13 receptor alpha1 (IL-13Ralpha1) signaling regulates intestinal epithelial cystic fibrosis transmembrane conductance regulator channel-dependent Cl- secretion. J Biol Chem.

[41] Peng XD, Xu PZ, Chen ML, Hahn-Windgassen A, Skeen J, Jacobs J, Sundararajan D, Chen WS, Crawford SE, Coleman KG, Hay N (2003). Dwarfism, impaired skin development, skeletal muscle atrophy, delayed bone development, and impeded adipogenesis in mice lacking Akt1 and Akt2. Genes Dev.

[42] Easton RM, Cho H, Roovers K, Shineman DW, Mizrahi M, Forman MS, Lee VM, Szabolcs M, de Jong R, Oltersdorf T, Ludwig T, Efstratiadis A, Birnbaum MJ (2005). Role for Akt3/protein kinase Bgamma in attainment of normal brain size. Mol Cell Biol.

[43] Bae SS, Cho H, Mu J, Birnbaum MJ (2003). Isoform-specific regulation of insulin-dependent glucose uptake by Akt/protein kinase B. J Biol Chem.

[44] Irie HY, Pearline RV, Grueneberg D, Hsia M, Ravichandran P, Kothari N, Natesan S, Brugge JS (2005). Distinct roles of Akt1 and Akt2 in regulating cell migration and epithelial-mesenchymal transition. J Cell Biol.

[45] Calera MR, Martinez C, Liu H, Jack AK, Birnbaum MJ, Pilch PF (1998). Insulin increases the association of Akt-2 with Glut4-containing vesicles. J Biol Chem.

[46] Kupriyanova TA, Kandror KV (1999). Akt-2 binds to Glut4-containing vesicles and phosphorylates their component proteins in response to insulin. J Biol Chem.

[47] Yang ZZ, Tschopp O, Hemmings-Mieszczak M, Feng J, Brodbeck D, Perentes E, Hemmings BA (2003). Protein kinase B alpha/Akt1 regulates placental development and fetal growth. J Biol Chem.

[48] Okano J, Gaslightwala I, Birnbaum MJ, Rustgi AK, Nakagawa H (2000). Akt/protein kinase B isoforms are differentially regulated by epidermal growth factor stimulation. J Biol Chem.

[49] Maroulakou IG, Oemler W, Naber SP, Tsichlis PN (2007). Akt1 ablation inhibits, whereas Akt2 ablation accelerates, the development of mammary adenocarcinomas in mouse mammary tumor virus (MMTV)-ErbB2/neu and MMTV-polyoma middle T transgenic mice. Cancer Res.

[50] Cristiano BE, Chan JC, Hannan KM, Lundie NA, Marmy-Conus NJ, Campbell IG, Phillips WA, Robbie M, Hannan RD, Pearson RB (2006). A specific role for AKT3 in the genesis of ovarian cancer through modulation of G(2)-M phase transition. Cancer Res.

[51] Cheng JQ, Ruggeri B, Klein WM, Sonoda G, Altomare DA, Watson DK, Testa JR (1996). Amplification of AKT2 in human pancreatic cells and inhibition of AKT2 expression and tumorigenicity by antisense RNA. Proc Natl Acad Sci U S A.

[52] Rychahou PG, Kang J, Gulhati P, Doan HQ, Chen LA, Xiao SY, Chung DH, Evers BM (2008). Akt2 overexpression plays a critical role in the establishment of colorectal cancer metastasis. Proc Natl Acad Sci U S A.

[53] Davies MA, Stemke-Hale K, Tellez C, Calderone TL, Deng W, Prieto VG, Lazar AJ, Gershenwald JE, Mills GB (2008). A novel AKT3 mutation in melanoma tumours and cell lines. Br J Cancer.

[54] Ben-Sahra I, Howell JJ, Asara JM, Manning BD (2013). Stimulation of de novo pyrimidine synthesis by growth signaling through mTOR and S6K1. Science.

[55] Lee CC, Huang HY, Chiang BL (2011). Lentiviral-mediated interleukin-4 and interleukin-13 RNA interference decrease airway inflammation and hyperresponsiveness. Human Gene Therapy.

